# Beef quality parameters estimation using ultrasound and color images

**DOI:** 10.1186/1471-2105-16-S4-S6

**Published:** 2015-02-23

**Authors:** Jose Luis Nunes, Martín Piquerez, Leonardo Pujadas, Eileen Armstrong, Alicia Fernández, Federico Lecumberry

**Affiliations:** 1 Departamento de Procesamiento de Señales, Instituto de Ingeniería Eléctrica, Facultad de Ingeniería, Universidad de la República, Montevideo, Uruguay; 2 Departamento de Genética y Mejora Animal, Facultad de Veterinaria, Universidad de la República, Montevideo, Uruguay

**Keywords:** beef quality, ribeye, backfat, intramuscular fat, ultrasound images, curve evolution, feature extraction, support vector regression

## Abstract

**Background:**

Beef quality measurement is a complex task with high economic impact. There is high interest in obtaining an automatic quality parameters estimation in live cattle or post mortem. In this paper we set out to obtain beef quality estimates from the analysis of ultrasound (in vivo) and color images (post mortem), with the measurement of various parameters related to tenderness and amount of meat: rib eye area, percentage of intramuscular fat and backfat thickness or subcutaneous fat.

**Proposal:**

An algorithm based on curve evolution is implemented to calculate the rib eye area. The backfat thickness is estimated from the profile of distances between two curves that limit the steak and the rib eye, previously detected. A model base in Support Vector Regression (SVR) is trained to estimate the intramuscular fat percentage. A series of features extracted on a region of interest, previously detected in both ultrasound and color images, were proposed. In all cases, a complete evaluation was performed with different databases including: color and ultrasound images acquired by a beef industry expert, intramuscular fat estimation obtained by an expert using a commercial software, and chemical analysis.

**Conclusions:**

The proposed algorithms show good results to calculate the rib eye area and the backfat thickness measure and profile. They are also promising in predicting the percentage of intramuscular fat.

## Background

**Figure 1:**
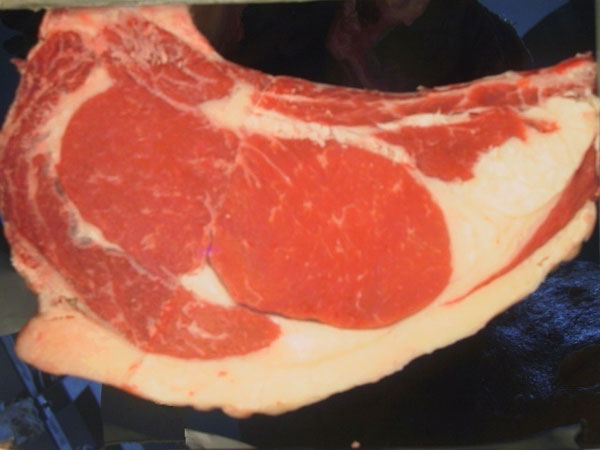
**Sample RGB image**. Sample image acquired at the slaughterhouse using a special hardware controlling distance and lighting.

In meat industry it is critical to have objective indicators that measure beef quantity and quality. It is also desirable that these indicators are non-destructive and fast to compute. Uruguay produces about 550,000 tons of beef a year, 30% for domestic consumption and the rest for export. One reason for the high production levels and acceptance in the world is the implementation of a good traceability and information systems. In this context, it is very important to have efficient methods to estimate parameters related with meat quality and amount of beef: rib eye area, percentage of intramuscular fat (IMF%) in the rib eye and subcutaneous backfat and rumb fat thickness. Obtaining predictors from ultrasound or color images (Figure 1) fulfill all these goals.

**Figure 2:**
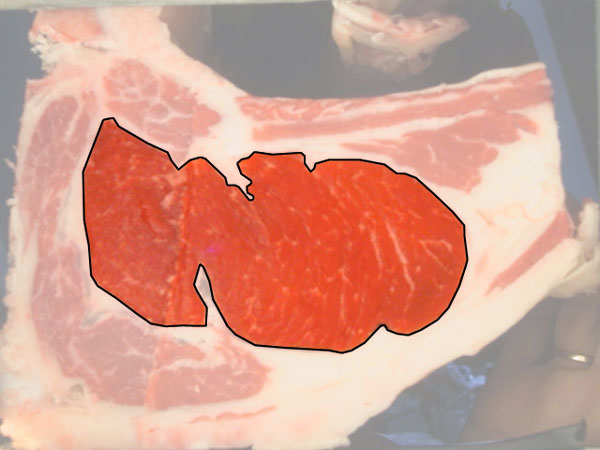
**Sample RGB image**. Backfat region selected.

**Figure 3:**
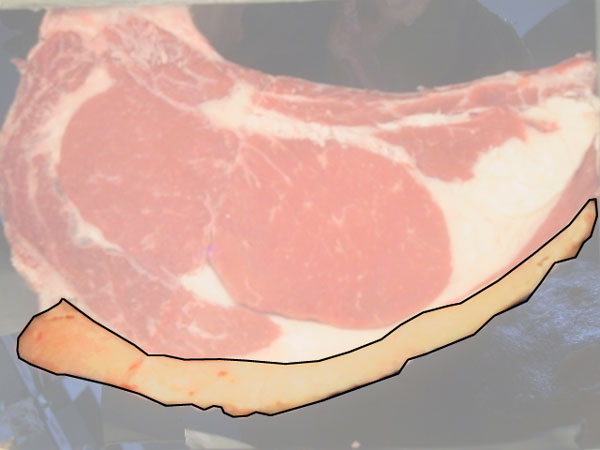
**Sample RGB image**. Backfat region selected.

The rib eye area (Figure 2) is an important parameter since it allows to estimate the degree of carcass yield, as it is linked to the amount of meat or muscle with high impact in its price [1]. Also, having a well distributed, and in a narrow ratio, fat coverage is considered a desirable attribute. Traditionally, these indicators are obtained through manual procedures performed by experts at the meat processing plants [2]. For example, an usual way to measure subcutaneous fat thickness (Figure 3) is performed manually, using a ruler.

Such procedures are done in inhospitable environments and consist of repetitive tasks that are tedious for the expert, with high error rates linked to fatigue and inspector's mood. In [3,4], methods for automatic segmentation of the rib eye in color images are proposed. These methods separate meat from "non-meat" (fat and bones). However, this method treats equally all the meat in the image, since the rib eye is not always surrounded by "non-meat" and includes other adjacent muscles in detection. Some works [4], avoid this problem removing other regions of the steak leaving only the rib eye beef; clearly this method is not suited for evaluating carcass at the slaughterhouse. In this work we propose a method based on curve evolution both for rib eye area and subcutaneous fat thickness measurements.

**Figure 4:**
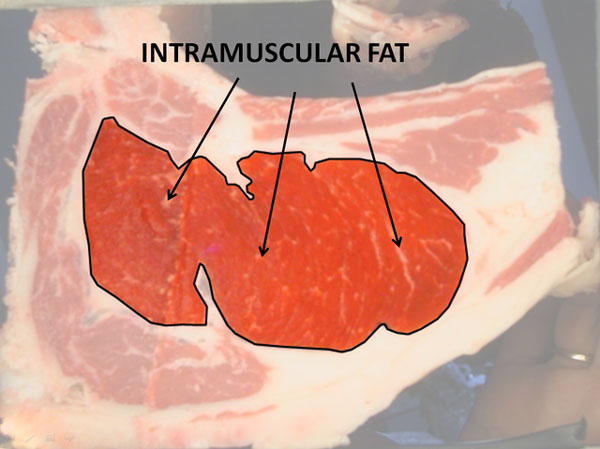
**Sample RGB image**. Intramuscular fat.

The intramuscular fat percentage (IMF%) is the proportion of intramuscular fat in the rib eye (Figure 4). IMF% is highly correlated with organoleptic characteristics such as juiciness and flavour [5], and above all it is a determining factor in perception of tenderness, this being the indicator with the highest impact on meat quality. It is for this reason that its estimation is essential in order to contribute to the carcass categorization. This quality indicator is usually performed in slaughtered animals, however, the indicator would also be of value if it could be measured in living animals for purposes of selective feeding, breeding and rearing [6]. For that reason it is becoming important to develop automatic measurements and analysis algorithms both on color images (post mortem) and ultrasound images (ante morten) in livestock.

There are several previous work in this kind of applications, such as [7,8] addressing the estimation of the IMF% in ultrasound images for livestock. In [9] the rib area was used as a determinant factor in the estimation of beef quality.

The production method used in Latin America usually includes a high component of extensive farming (although feedlot is used too) impacting in the amount of IMF%, while in other regions the feedlot production is preferred [10,6,11]. Therefore, IMF% in animals analysed in previous works such as [12,6,7,13] might be different from the animals analysed in the present work and predictors should be adjusted in this case.

In this work we present new methods to automatically measure three beef quality parameters, IMF% in ultrasound images, rib eye area and backfat thickness in color images in slaughtered steers.

## Automatic measurement methods

This section describes the proposed methods for ribeye detection and area estimation, then we present the method for subcutaneous fat detection which allows to estimate the thickness and to extract the fat profile. Finally the proposed strategy for IMF% is presented.

### Rib eye detection and area estimation

The rib eye area estimation is used as a quality indicator and is also used for carcass categorizing the animal. Database images show a cross-sectional area of the *longissimus dorsi *muscle (dorsal length) at the level of the 12th intercostal space. The objective is to estimate the area of the muscle, commonly measured manually by an expert [5,2].

This works proposes a curve evolution algorithm, more precisely an adaptation of the algorithm *Distance Regularized Level Set Evolution *(DRLSE)[14]. This method is a recent improvement of the *level set *techniques [15,16]. The DRLSE method adds an intrinsic capability to the level set framework for maintaining regularity of the level set function, particularly maintaining the property of signed distance function around the zero level set. This method is used in an edge-based active contour segmentation that modifies the geodesic active contour proposed by Caselles [17]. Therefore, edge information computed from the image is used to create the energy functional that govern the level set evolution.

In our framework, this information is provided using two different energy terms computing information from the image's edges, one uses "high contrasted" edge information and the other uses "emphasized" edge information. "High contrasted" edges are computed using the Caselles equation (1) on the binary image result of the "non-meat" detection. "Emphasized" edges are computed using Caselles equation but now in the *u *color channel, after an anisotropic diffusion on the image.

(1) $$g≜\frac{1}{1+{\left|\nabla G_{\sigma }*I\right|}^{2}}$$

where *G_σ _*is the Gaussian kernel with the standard deviation *σ*.

#### Preprocessing

**Figure 5:**
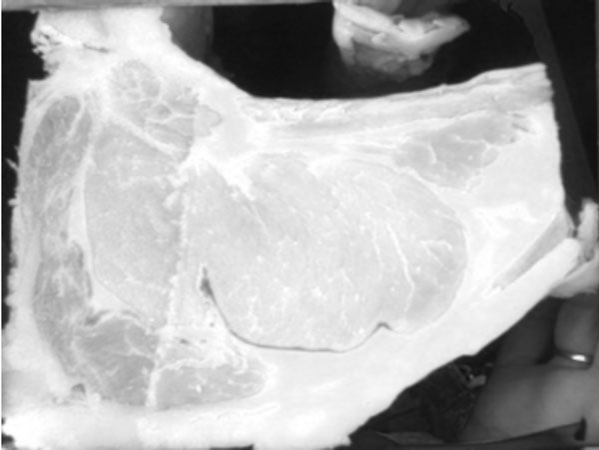
**Background elimination**. Red channel from de RGB space.

**Figure 6:**
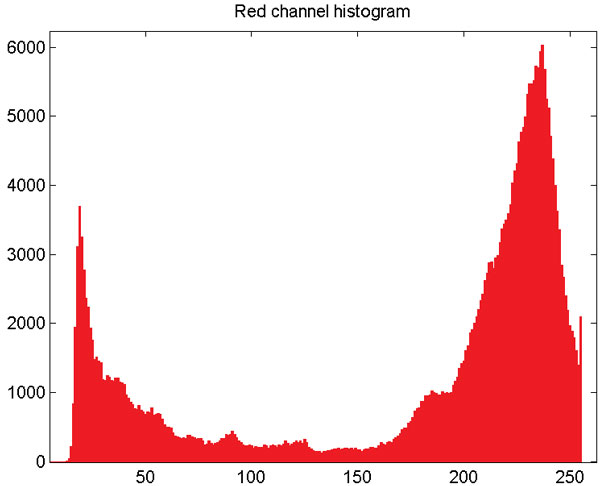
**Background elimination**. Histogram from the red channel.

**Figure 7:**
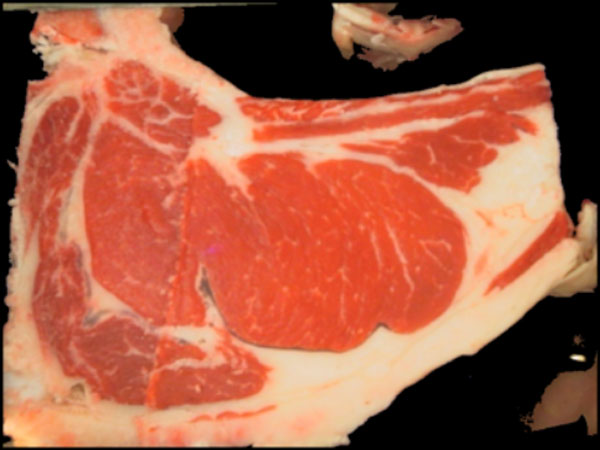
**Background elimination**. Final step.

**Figure 8:**
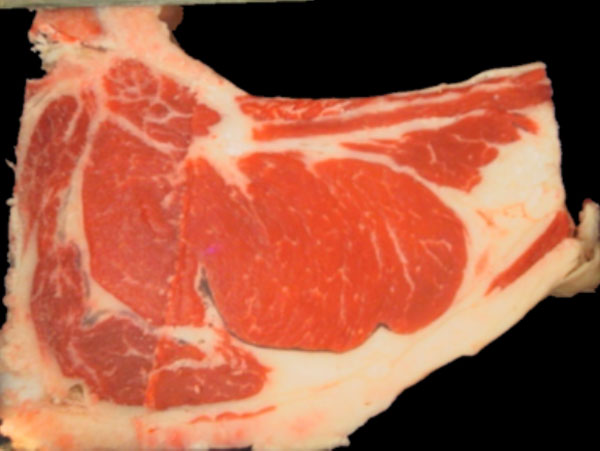
**Background elimination**. Result of the background elimination.

A preprocessing stage which includes background elimination and detection of the "meat" and "non-meat" regions is done before the curve evolution stage. The background elimination is performed starting with an Otsu's binarization [18] on the red channel of color (Figure 5 shows the red channel). This procedure assumes there are two classes of pixels' intensities and looks for the optimal threshold that minimizes the intra-class variance. Regions in the steak (meat, bones and fat) contains enough information in the red channel to separate it from the background, which is majority black (Figure 6). Small gaps in the background are filled and the background is removed as shown in Figure 7 and 8.

**Figure 9:**
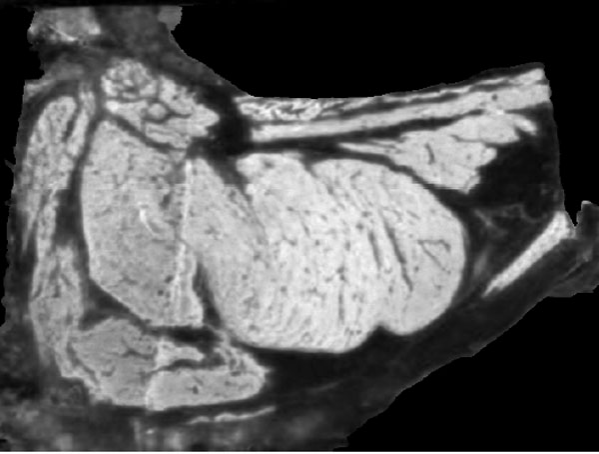
**"Meat" and "non meat" detection**. U channel from de Luv space.

**Figure 10:**
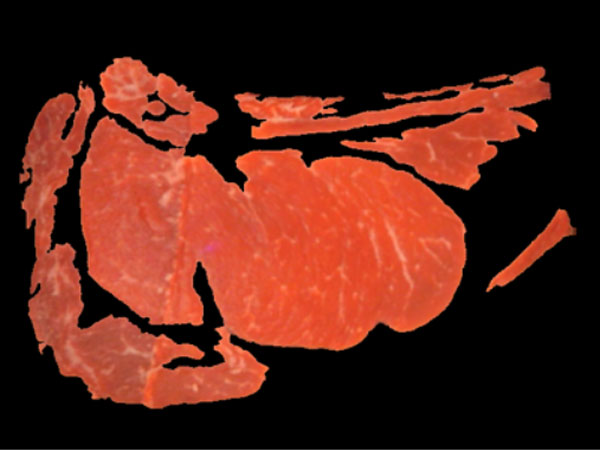
**"Meat" and "non meat" detection**. Meat regions.

**Figure 11:**
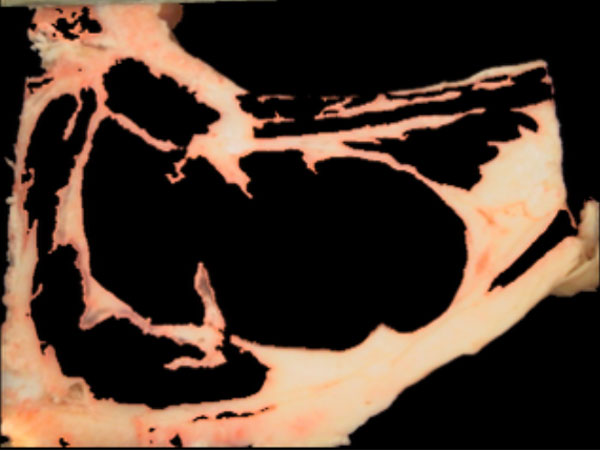
**"Meat" and "non meat" detection**. Non-meat regions.

For the detection of the "meat" and "non-meat" regions the information on the *u *(uniformity) (Figure 9) and *L *(luminance) channels in the *Luv *color space, and the G (green) channels inthe RGB color space are binarized, unsing Otsu's method, and their results combined. These color channels exhibit a good discrimination between "meat" and "non-meat" regions. Figures 10 and 11 show the result of this step.

#### Curve evolution

**Figure 12:**
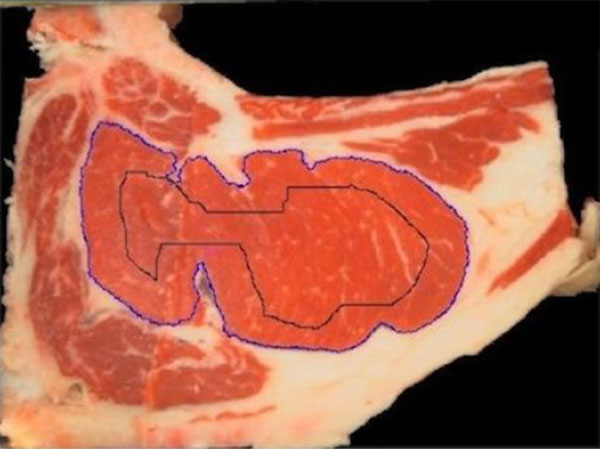
**Curve evolution steps**. Initial curve.

**Figure 13:**
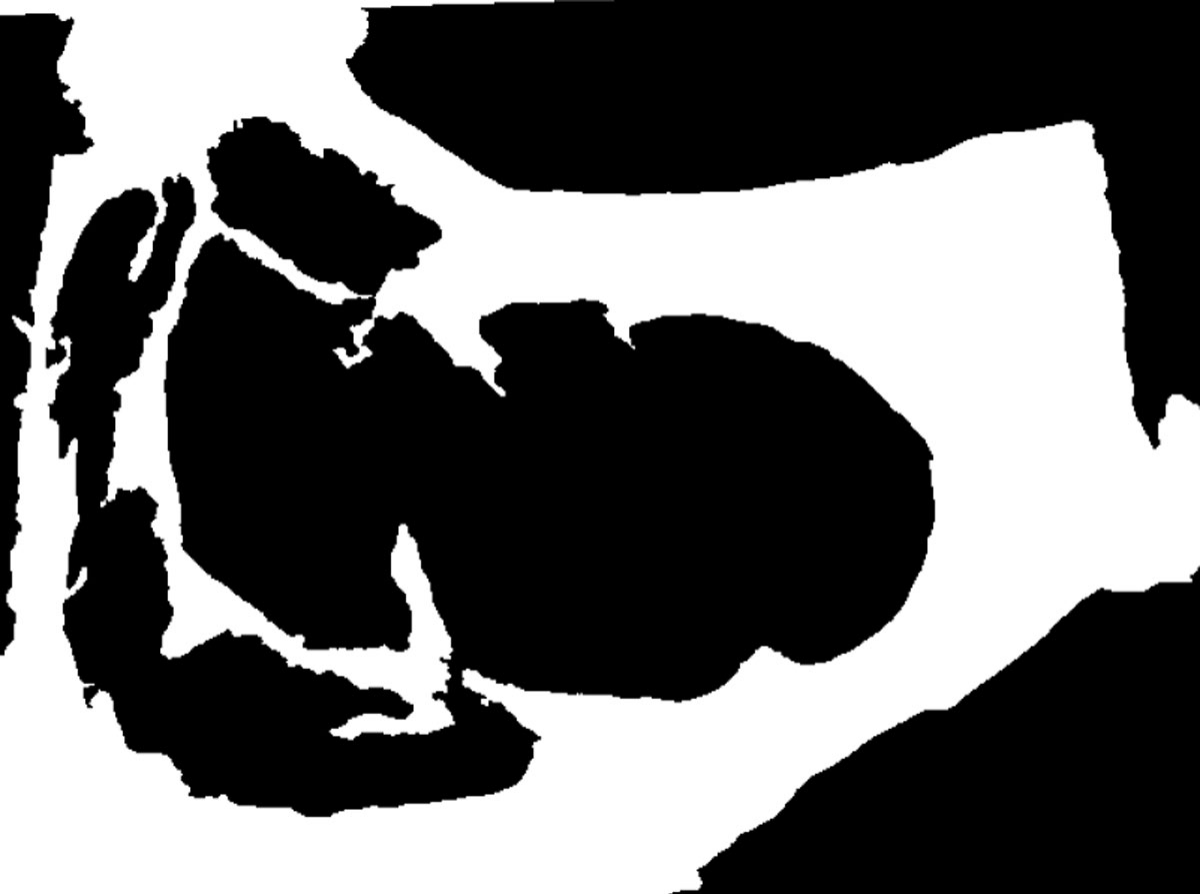
**Curve evolution steps**. Steps curve evolution.

**Figure 14:**
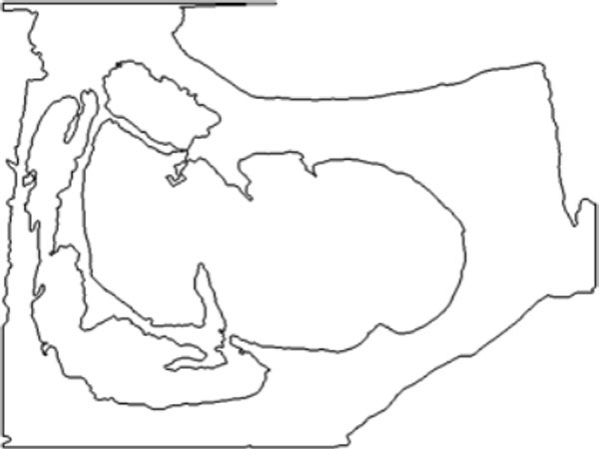
**Curve evolution steps**. High contrasted edges.

**Figure 15:**
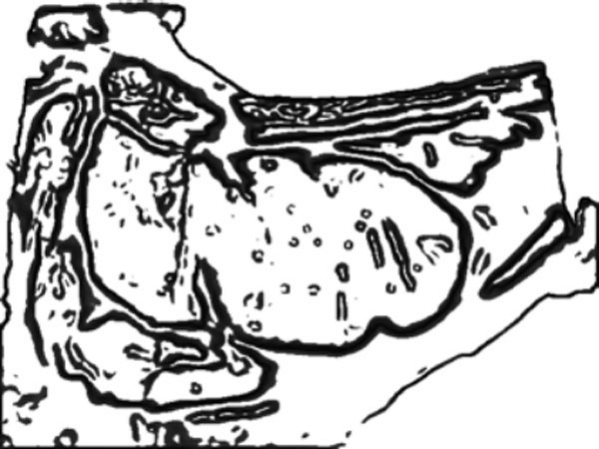
**Curve evolution steps**. Emphasized edges.

Curve evolution is performed in three stages, starting from an initial curve determination. Image erosion is applied on the image result of the meat segmentation and with the consequent elimination of small regions of meat. This results in an internal region into the muscle of interest, Figure 12. Since the rib eye is the biggest meat region present in the steak, the erosion is performed iteratively until only one connected component is present in the image that should belong to the interior of the rib eye. The edges of this region defines the initial curve, that in the first stage of the curve evolution algorithm is evolved by considering only the role of the "high-contrasted" edges (Figure 13,14,15). This evolution seeks to achieve an initial approach to the contour of the rib eye. This evolution is fast, looking for reducing the segmentation time, specially in cases in which the initial contour is far from the rib eye contour. In the second stage, the algorithm continues from the previous evolution adding the "emphasized" edges energy term. This evolution is slower and stops when the growth area between successive evolutions does not exceed certain learned threshold.

In general, the adjustment of the curve to the rib eye edges achieved in these two steps is quite accurate. However, a third stage of evolution adjustment is applied, performing ten more iterations in order to achieve finer adjustment.

### Backfat measurement

Measurement of the backfat thickness in color images is made on the cross section at the level of twelfth intercostal space, perpendicular to the outer edge of the fat and up to a quarter of the end of the muscle *longissimus dorsi *respect to the backbone. Fat provides desirable attributes, a well distributed coverage associated with a creamy-white color, are considered ideal; however excessive amounts of fat must be removed (industrial process called "conditioning"), which significantly decreases meat yield.

**Figure 16:**
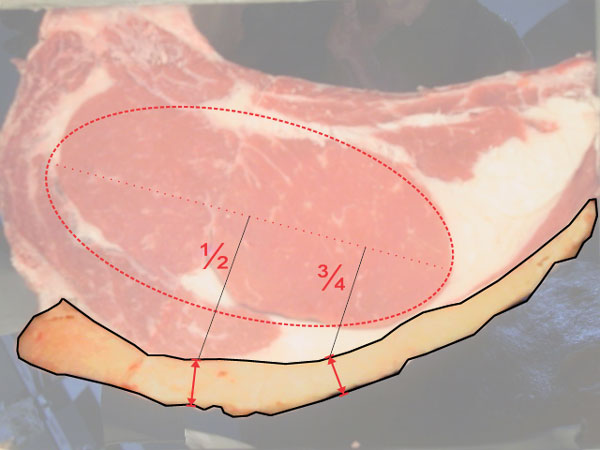
**Backfat segmentation**. Backfat marks 1/2 and 3/4.

Nowadays most of the measures are performed manually by an expert using a ruler. Backfat measures are taken in two points, corresponding to the projection of 1/2 and 3/4 lenghts of the axis of the rib eye's first moment (See Figure 16) [2].

The proposed method automatically determines the axis of the rib eye's first moment and measures the thickness in the corresponding points. Moreover, the method computes a full profile of thickness for all the backfat, allowing to measure the thickness along it. The algorithm is based in a curve evolution from an initial curve to a target curve, assigning a thickness proportional to the iterations needed in each point to reach the target curve.

It needs two closed curves whose intersection is empty and the backfat is between. These curves will be the input of the curve evolution algorithm. To provide good detection, the initial curve should match the inner boundary of the backfat and a section of the target curve must match the outer limit of the backfat.

**Figure 17:**
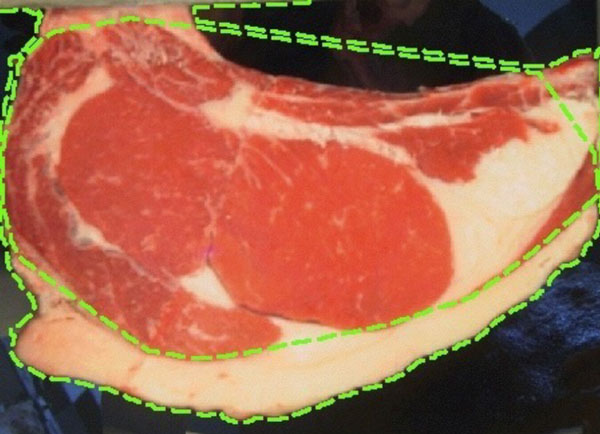
**Backfat segmentation**. Initial and target curves.

Backfat is characterized by not containing meat and to be part of the edge of the steak. Therefore, the initial curve is the convex hull of the beef meat. The target curve will be the edge of the union of the region defined after the background elimination and the region defined by the initial curve (see Figure 17). On the contact points a dilatation is performed over the target curve to avoid contact between both curves.

As said before, backfat should be measured in two points given by the perpendicular projection of half and 3/4 lengths of rib eyes's main axis. This point are geometrically located from the rib eye segmentation, computing its main axis.

#### Curve evolution

**Figure 18:**
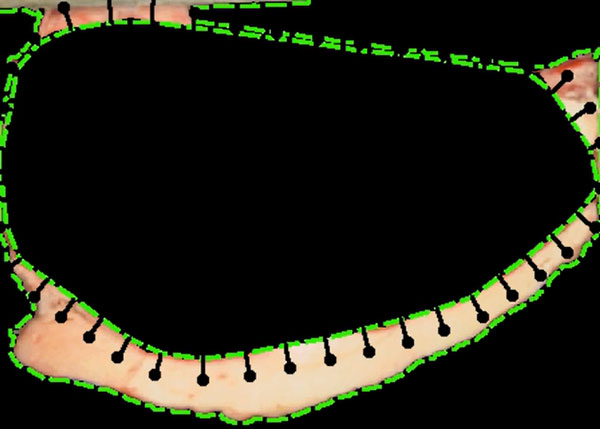
**Backfat segmentation**. Backfat curves evolution.

The evolution is based on the normal evolution of the interior curve (the initial curve) and considers the other curve as target. The evolution includes some mechanisms for creating and combining points to ensure a correct sampling at all steps [19]. The thickness is measured as the length of the path in the evolution. This algorithm finds an intuitive correspondence between points of the different curves and leads to an appropriate thickness definition. Since in this case there is no need of lot of points, the initial curves are resampled every 50 points. This has a big impact on the execution time generating a fast evolution. Figure 18 shows the evolution in an intermediate step.

**Figure 19:**
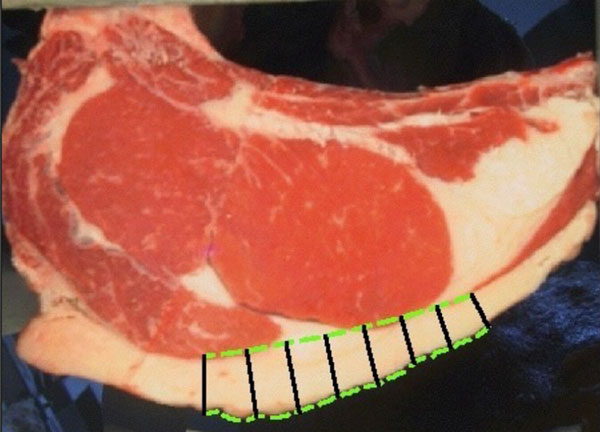
**Backfat segmentation**. Backfat result of segmentation.

Finally the region of interest between the measures 1/2 and 3/4 is taken as shown in Figure 19.

### Intramuscular fat percentage estimation

Intramuscular fat percentage (IMF%) is the proportion of fat between the muscle fibers of the rib eye. This quality measure can be performed in color images in slaughtered animals, and in ultrasound images in live animals. It has been shown that intramuscular fat percentage is highly correlated with tenderness [20,2], which is highlighted for consumers as one of the most determinant factors in beef quality. Therefore an automatic system for its measurement results fundamental.

In this work we propose its measure through two types of images, color images and ultrasound images.

The algorithm proposed includes three stages: region of interest detection, features extraction and selection and modelling for the estimation.

#### Region of interest detection

**Figure 20:**
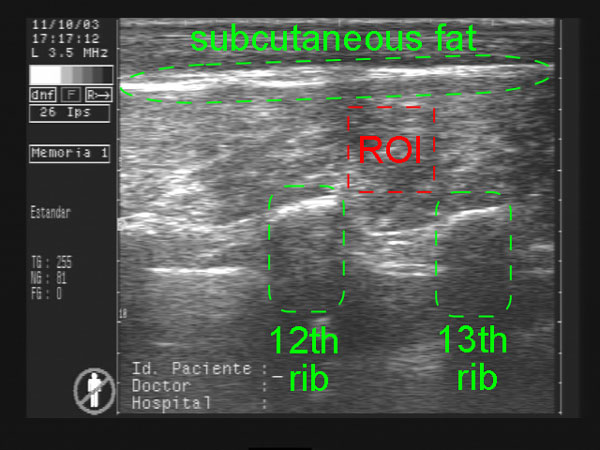
**IMF% estimation on ultrasound images**. Sample US image.

Only the muscle between the 12th and 13th rib under the subcutaneous backfat is taken to determine the IMF% value. Therefore, the region of interest (ROI) is defined given these structural components present in the ultrasound images, see Figure 20. The ultrasound image acquisition guarantees that all these three components are present.

**Figure 21:**
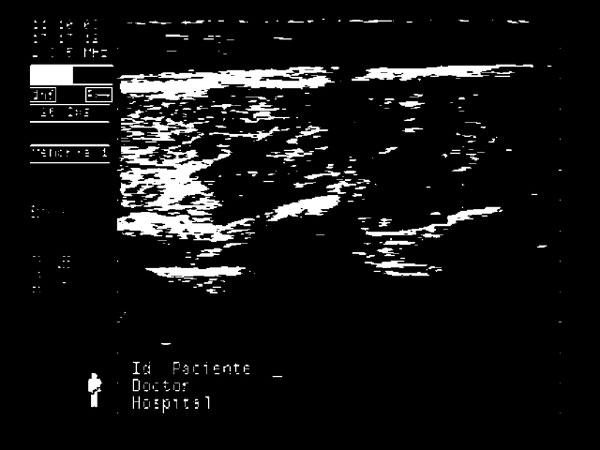
**IMF% estimation on ultrasound images**. Otsu's processed image.

For the subcutaneous fat location a thresholding using Otsu's method is applied for binarizing the image (see Figure 21) and a labelling algorithm was run on the image in order to determine the different regions. Thus, the subcutaneous backfat region is defined as the labelled region with a shape like the subcutaneous fat, i.e., a thin, long horizontal region in the upper third part of the image. Therefore, the labeled object with the highest ratio between the horizontal and vertical length was set as the subcutaneous fat.

**Figure 22:**
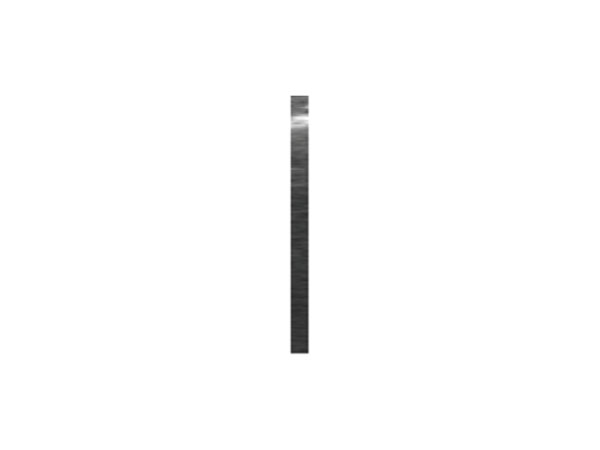
**IMF% estimation on ultrasound images**. Rib template.

For defining the location of the ribs, first, an anisotropic diffusion is applied to the image in order to smooth it without losing the edge information and restricting small variations of intensity in a same region. Then, a correlation between the image and a synthetic template emulating a generic rib (see Figure 22) was performed. The two local maxima in magnitude found in the correlation result represent the location of the ribs.

**Figure 23:**
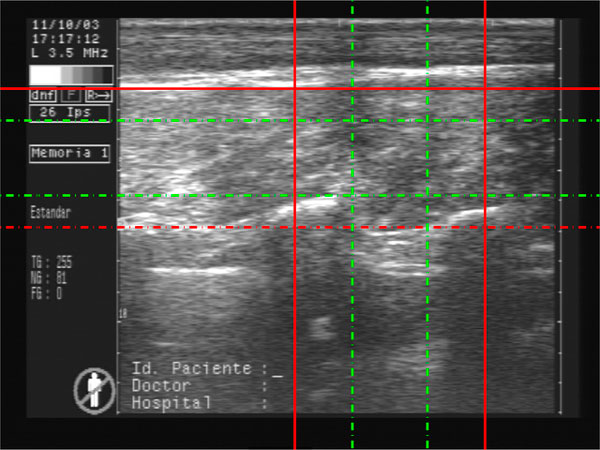
**IMF% estimation on ultrasound images**. Output image.

Finally, the ROI is defined as a 80 × 80 pixels square set on the center zone delimited by the subcutaneous fat and the ribs. Figure 23 shows an example of the output from the ROI detection procedure.

#### Features extraction and selection

A set of forty two features were extracted from each ROI. These features are based on several statistics and transformation on the ROI, for example, texture descriptors, local binary pattern, co-occurrence matrix, histograms, Fourier Transform, etc. Table 1 summarizes the computed features.

**Table 1:** Features extracted from the US images

Gradient
- mean µ* - std deviation σ*

**Co-occurrence matrix**

- correlation* - homogeneity* - contrast* - energy*

**Gray Level**

- mean - contrast ratio

**Histogram**

- percentile (each 20%) - skewness

**Fourier transform**

- variance coefficient - power percentile (× 5)

**Local Binary Pattern (LBP)**

- correlation - homogeneity - contrast - energy

Features marked with * were computed in four directions given by angles (45°,90°,135°,180°)

As a result of the feature acquisition stage we obtain a 42-dimension feature space. To reduce the space dimension in order to improve computational performance a feature selection stage based on Principal Components Analysis (PCA) was done, finding that 99% of the variance is accumulated in the first ten components. As a result of the PCA a new space of ten new features, linear combinations of the original ones, was used to do the IMF% estimation model.

#### Modeling for the estimation

Support Vector Regression (SVR) is a variant of the classic Support Vector Machine algorithm. The basic idea of SVR consist in mapping the training data, x∈X, into a larger dimensional space  via a nonlinear mapping Φ: X→F, where a linear regression can be performed. For more details on SVR see [21,22].

In this work, a radial basis function $\left(g\left(u,v\right)=e^{-\gamma {\left|u-v\right|}^{2}}\right)$) was used as kernel. Parameters *γ *and tolerance of termination criterion were optimized based on the data train set.

The same strategy was applied for the post mortem color images, using the rib eye area as the ROI. Similar performance was obtained using specific descriptors for color data (intensity mean in different color channels, Fourier coefficients, number of pixels in each channel).

## Experiments and results

### Database

We worked with a database of 153 steers acquired at the slaughter using a special hardware controlling distance and lighting. Only for 71 of them having ultrasound images. Ultrasound images were collected at a cattle ranch in Uruguay.

A measure of the rib eye area performed by an expert for all the database was used for the validation process, as well as a measure of backfat 1/2 and 3/4 for 51 steers.

IMF% was measured by chemical gas chromatography analysis and used as *ground truth *to validate the regression results. The lipid extraction protocols used are described in [23]; its margin of error in the measurement is less than 0.3%. An estimation of the IMF% from ultrasound images was performed by an expert from the meat industry using a commercial software. 283 ultrasound images were acquired, four images were taken per animal. The ultrasound hardware used was the *Aquila Pro Vet*, an industry standard equipment.

### Rib eye area

The database was divided into two sets randomly drawn, one to train the algorithm and the parameters optimization (103 images, 2/3 of the dataset) and the other to test it (50 images, 1/3 of the dataset).

Two performance indicators were used,

• *Relative area error:*

(2) $$\epsilon_{1}=\frac{\left|A_{auto}-A_{manual}\right|}{A_{manual}}$$

• *Concordance:*

(3) $$\epsilon_{2}=1-\frac{A_{inter}}{A_{union}}$$

where *A_union _*= *R_auto _*∪ *R_manual _*y *A_inter _*= *R_auto _*∩ *R_manual_*. With *R_auto _*rib eye area automatically detected by the implement algorithm and *R_manual _*rib eye area measured by an expert manually.

The results obtained for images rib eye segmentation are showed in Tables 2 and 3,

**Table 2:** Results for the rib eye area estimation for images with errors less than 10%.

	Size	ϵ_1_	ϵ_2_
Train	103	86.4%	76.7%
Test	50	82.0%	78.0%

**Table 3:** Results for the rib eye area estimation for images with errors less than 15%.

	Size	ϵ_1_	ϵ_2_
Train	103	89.3%	86.4%
Test	50	90.0%	88.0%

### Backfat

For the Backfat measure an estimation of the 1/2 and 3/4 is automatic done using a database of 51 steer and then contrasted with the expert measure. Table 4 compares the result of the algorithm developed and the expert measure.

**Table 4:** Results for the backfat thickness estimation.

	1/2	3/4
RMSE	8.7	7.6
R2	0.87	0.88

**Figure 24:**
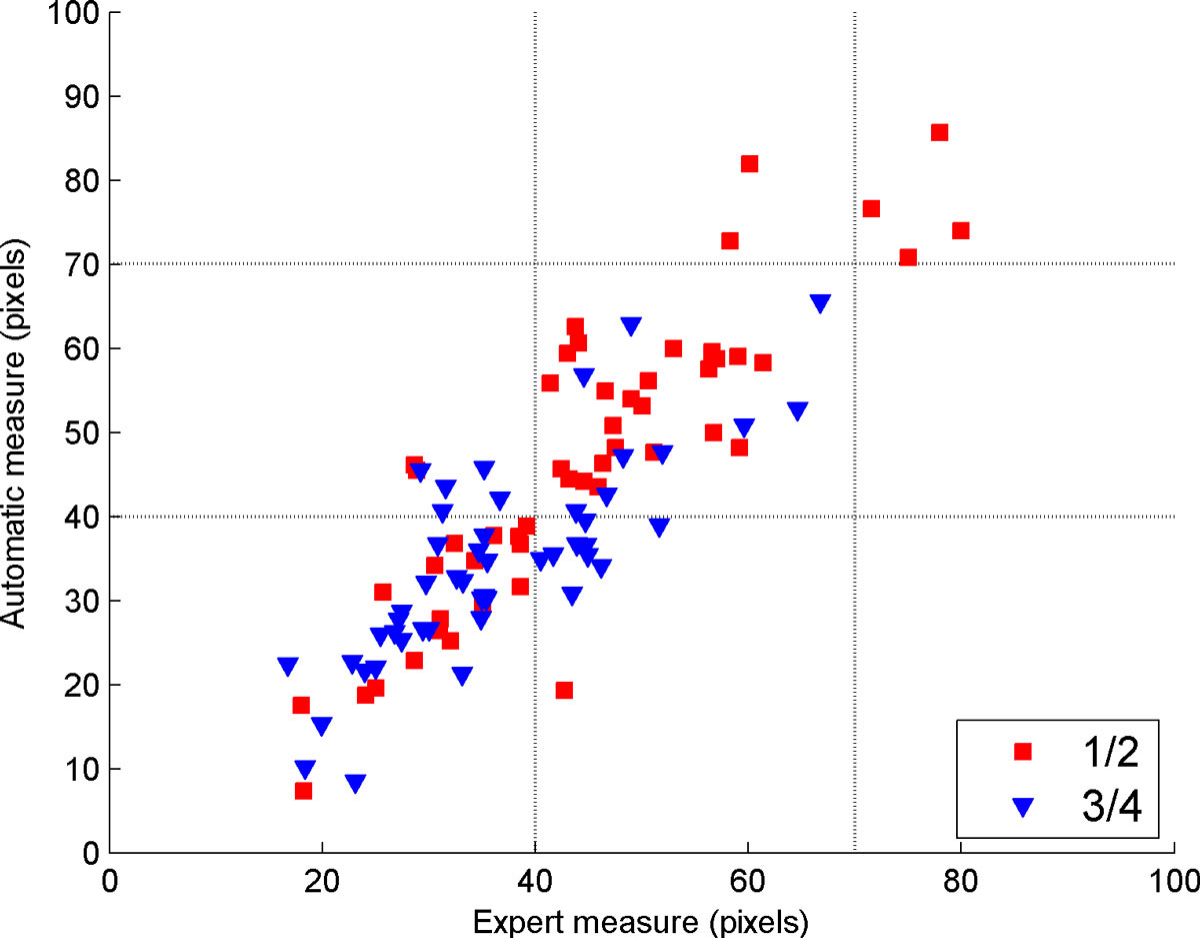
**Backfat estimation algorithm**. Scatter plot of the backfat estimation algorithm developed and the ground truth obtained by an expert measure. The 51 animals are represented in the graphic.

The results were *RMSE *= 8.7 and 7.6 (in pixels) respectively for 1/2 and 3/4 and *R*2 = 0.87 and 0.88, where *RMSE *is the root mean square and *R*2 is the Pearson coefficient of correlation. Figure 24 shows the 51 animals in a scatter plot of the Backfat measure vs. the expert measure.

### Intramuscular fat percentage

The database of ultrasoud images was divided into two sets randomly drawn, one to train the algorithm and compute the linear regression coefficients (184 images, 2/3 of the dataset) and the other to test it (92 images, 1/3 of the dataset).

**Figure 25:**
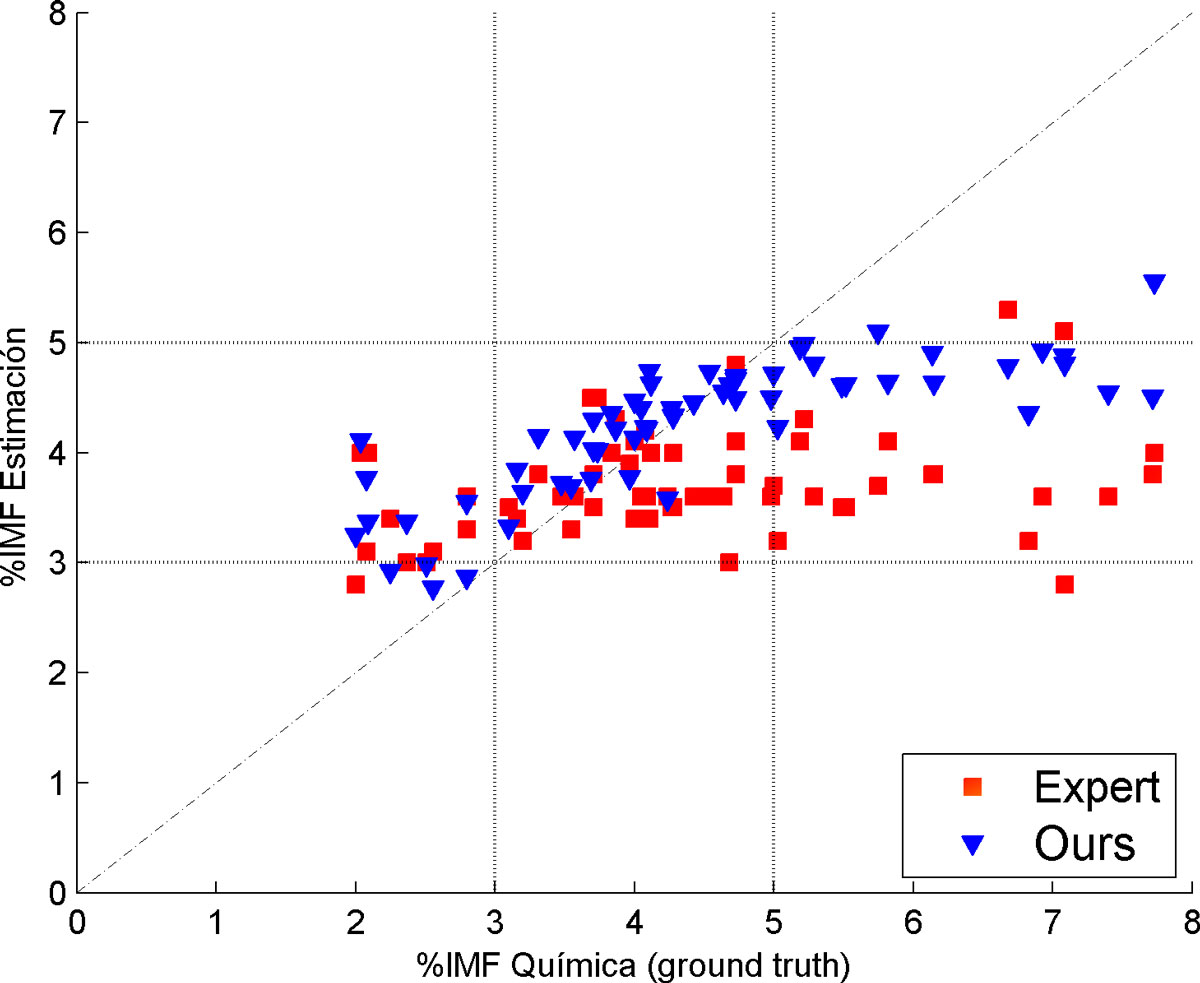
**IMF% results in ultrasound images**. IMF% results in ultrasound images.

This procedure was repeated 100 times, varying the test and training set. The results were: *RMSE *= 1.31 and *R*2 = 0.37. Figure 25 shows the 71 animals in a scatter plot of the IMF% estimation vs. the *ground truth*. To contrast, the estimation of the IMF% made by the expert, which has an RMSE of 1.58 and a correlation coefficient of 0.23. Table 5 compares the result of the algorithm developed and the expert estimation.

**Table 5:** Results for the IMF% estimation.

	Ours	Expert
RMSE	1.31	1.58
R2	0.37	0.23

## Conclusions and future work

New automatic methods to measure different beef quality parameters from the analysis of ultrasound (in vivo) and color images (post mortem) are presented. The parameters estimated are related to meat quality, meat yield and consumer's health: eye area steak, percentage of intramuscular fat and backfat thickness or subcutaneous fat.

First of all, we propose to measure the rib eye area, using an algorithm based on curve evolution. The results are satisfactory in over 77% of the processed images (seven different data sets with different acquisition conditions). The method is very robust and general, making it suitable for the segmentation of other muscle regions. In controlled conditions results are even better (more than 89%). Second, we propose to measure backfat thickness from the profile of distances between a curve that limits the steak and one that limits the rib eye previously detected. The automatic distance measurement achieves a very precise estimate using a novel evolution strategy. Beside this results, the algorithm gives the backfat thickness profiles, which provides information on the uniformity of the subcutaneous fat which is an important carcass quality information.

Last, we propose a procedure for estimating the intramuscular fat percentage based in Support Vector Regression in a region of interest automatically determined for ultrasound images (rectangle) and for the color images (rib eye). The performance of the automatic selection of the ROI for ultrasound was highly satisfactory, more than 96% of the database were well detected, in the remaining 4% where the ROI was wrongly detected, the software gives an alert and allows for a manual definition. The prediction of intramuscular fat showed a better adjustment only in the middle range of fat percentages (3%-5%). However this error in our approach is lower than the error in the expert's estimation. The overall performance is promising, clearly a deeper analysis of the features considered is needed. In the color images the main difference is in ROI determination and the set of features used, but we obtain similar performance results.

In future work we propose to analyse new strategies in the feature extraction and selection stages, both for ultrasound and color images, for intramuscular fat percentage estimation. We also want to explore the impact of different parameters in the estimation, such as the ROI's area and location.

## Competing interests

The authors declare that they have no competing interests.

## Authors' contributions

J.L.N., M.P. and L.P. carried out the implementation of the algorithms, performed the experiments, developed the analytical tools and wrote the first version of the manuscript. J.L.N. also participated in all the stages of the preparation and edition of the manuscript. E.A. created the database and participated in the review of the manuscript. A.F. and F.L. proposed algorithm, designed experiments, analysed data and wrote the paper. All authors discussed the results on the manuscript.

## Declarations

Publication costs for this article were funded by the Department of Signal Processing, Facultad de Ingeniería, Universidad de la República, Montevideo, Uruguay.

This article has been published as part of *BMC Bioinformatics *Volume 16 Supplement 4, 2015: Selected articles from the 9th IAPR conference on Pattern Recognition in Bioinformatics. The full contents of the supplement are available online at http://www.biomedcentral.com/bmcbioinformatics/supplements/16/S4.
